# Urban-Rural Disparities in Treatment and Outcomes for Colorectal Liver Metastases in the United States: A SEER-Medicare Analysis

**DOI:** 10.1245/s10434-026-20434-5

**Published:** 2026-08-28

**Authors:** Mayte Bryce-Alberti, Victoria Stoffel, Isabella Blanchard, Maansi Chalasani, Jack Scaife, Antoine Jeri-Yabar, Benjamin A. Bates, David G. Brauer, Charu Verma, Haejin In, Alexander A. Parikh, Hari S. Iyer, Brett L. Ecker

**Affiliations:** 1 Department of Surgery, Rutgers New Jersey Medical School, Newark, NJ USA; 2 Rutgers New Jersey Medical School, Newark, NJ USA; 3 Department of Surgery, Northwestern University Feinberg School of Medicine, Chicago, IL USA; 4 Department of Internal Medicine, Icahn School of Medicine at Mount Sinai Morningside/West, New York, NY USA; 5 Institute for Health, Health Care Policy, and Aging Research, Rutgers University, New Brunswick, NJ USA; 6 Section of Cancer Epidemiology and Health Outcomes, Rutgers Cancer Institute of New Jersey, New Brunswick, NJ USA; 7 Department of Medicine, Division of General Internal Medicine, Rutgers Robert Wood Johnson Medical School, New Brunswick, NJ USA; 8 Masonic Cancer Center, University of Minnesota Medical School, Minneapolis, MN USA

**Keywords:** Curative-intent liver-directed therapy, Geographic disparities, Multidisciplinary oncologic care, Survival analysis

## Abstract

**Background:**

Curative-intent resection of colorectal liver metastases (CRLM) offers an opportunity for long-term survival, yet access barriers correlated with area-level socioeconomic status (SES) and rurality may drive disparities in cancer care. Prior work shows lower liver-directed therapy use and survival in socioeconomically disadvantaged conditions, whereas the association between rurality and CRLM treatment and prognosis has not been previously studied.

**Methods:**

We conducted a retrospective population-based cohort study using the Surveillance, Epidemiology, and End Result-Medicare database (2000–2020) among patients aged 66–85 years with isolated CRLM. Urban-rural status was determined by using USDA Rural-Urban Commuting Area codes. Associations between rurality, receipt of therapy, and overall survival were evaluated by using multivariable logistic regression and Cox proportional hazards models, adjusting for patient and tumor characteristics.

**Results:**

Among 34,781 patients with isolated CRLM, 21.7% resided in rural areas. Rural patients were more likely to have rectal primary tumors (adjusted odds ratio [aOR] [95% confidence interval (CI)] 1.13 [1.04–1.24], *p* = 0.01) and synchronous metastatic disease (aOR [95% CI] 1.09 [1.02–1.17], *p* = 0.01). Rural residence was associated with higher odds of primary tumor resection (aOR [95% CI] 1.12 [1.02–1.22], *p* = 0.01), but lower odds of systemic chemotherapy (aOR [95% CI] 0.86 [0.81–0.92], *p* < 0.01) and curative-intent liver directed therapy (aOR [95% CI] 0.89 [0.80–0.99], *p* = 0.03). Rural residence was independently associated with worse overall survival (hazard ratio [HR] (95% CI) 1.08 [1.05–1.12], *p* < 0.01).

**Conclusions:**

Rural patients with CRLM were less likely to receive liver directed therapy and experienced inferior survival. These findings highlight persistent geographic disparities in cancer care delivery and outcomes.

**Supplementary Information:**

The online version contains supplementary material available at https://doi.org/10.1245/s10434-026-20434-5.

Colorectal cancer (CRC) is the third most commonly diagnosed malignancy and the third-leading cause of cancer-related mortality in the United States.^1^ Despite progress in screening, systemic therapy, and multidisciplinary management, CRC continues to impose a major burden on the national healthcare system, with approximately 154,270 new cases and more than 52,900 CRC-related deaths in 2025.^2^ Most deaths are attributable to colorectal liver metastases (CRLM), because the liver represents the predominant site of distant disease in more than 50% of all cases. Hepatic resection, ablation, and most recently, transplantation represents the standard curative therapies for CRLM and have yielded long-term survival for eligible patients.^3-5^

Barriers to access for curative-intent liver-directed therapy (LDT) may perpetuate inequities in cancer care delivery.^3^ Area-level disparity markers include both socioeconomic status and rurality. Several studies have demonstrated that race and socioeconomic status significantly influence utilization of LDT, with hepatic resection performed less frequently among Black patients and individuals with greater social vulnerability.^6–8^ Similarly, higher poverty rates are independently associated with lower rates of hepatic resection for CRLM. In contrast, the impact of rurality on CRLM treatment and prognosis has not been previously studied. It has been previously established that rural patients face persistent barriers to timely and oncologic treatment, including but not limited to disparities in screening accessibility and quality, staging accuracy, genomic testing, and treatment adherent to clinical guidelines.^9–11^ Given the limited availability of liver surgeons and specialized cancer care in rural regions,^12^ rural residence may represent an underrecognized determinant of treatment utilization and survival among patients with CRLM. This study aims to assess disparities in the treatments utilized and outcomes achieved in patients with CRLM according to their geographic residence across Surveillance, Epidemiology, and End Results (SEER)-contributing registries. By leveraging population-based data, this study aims to identify geographic barriers contributing to inequitable use of multidisciplinary oncologic treatment among CRLM patients.

## Methods

### Study Design, Data Source, Population, and Eligibility Criteria

A retrospective cohort study was performed by using the SEER-Medicare database from 2000–2020. This database integrates cancer registry information with Medicare Parts A and B claims. Patients aged 66–85 years diagnosed with colorectal adenocarcinoma were identified using the International Classification of Diseases for Oncology, Third Edition (ICD-0-3) topography, behavior and histology codes. CRLM were identified using a validated claims-based algorithm requiring ≥ 2 Medicare claims for secondary malignant neoplasm of the liver.^12,13^

Patients were required to have continuous Medicare Parts A and B enrollment for ≥ 12 months before CRLM diagnosis and could not be enrolled in a Medicare managed care plan during the study period. Patients with extrahepatic metastatic disease were identified using site-specific Medicare claims for secondary malignancies involving nonhepatic metastatic sites and were excluded. Consistent with prior SEER-Medicare studies of CRLM, patients were excluded if extrahepatic disease was diagnosed prior to curative-intent LDT or, if untreated, within 12 months of CRLM diagnosis to reduce misclassification of patients with initially unrecognized extrahepatic disease. The date of CRLM diagnosis served as the index date.^12,13^ Patients with unknown RUCA classification were excluded from the crude and multivariable analyses to maintain a binary comparison.

**Fig. 1:**
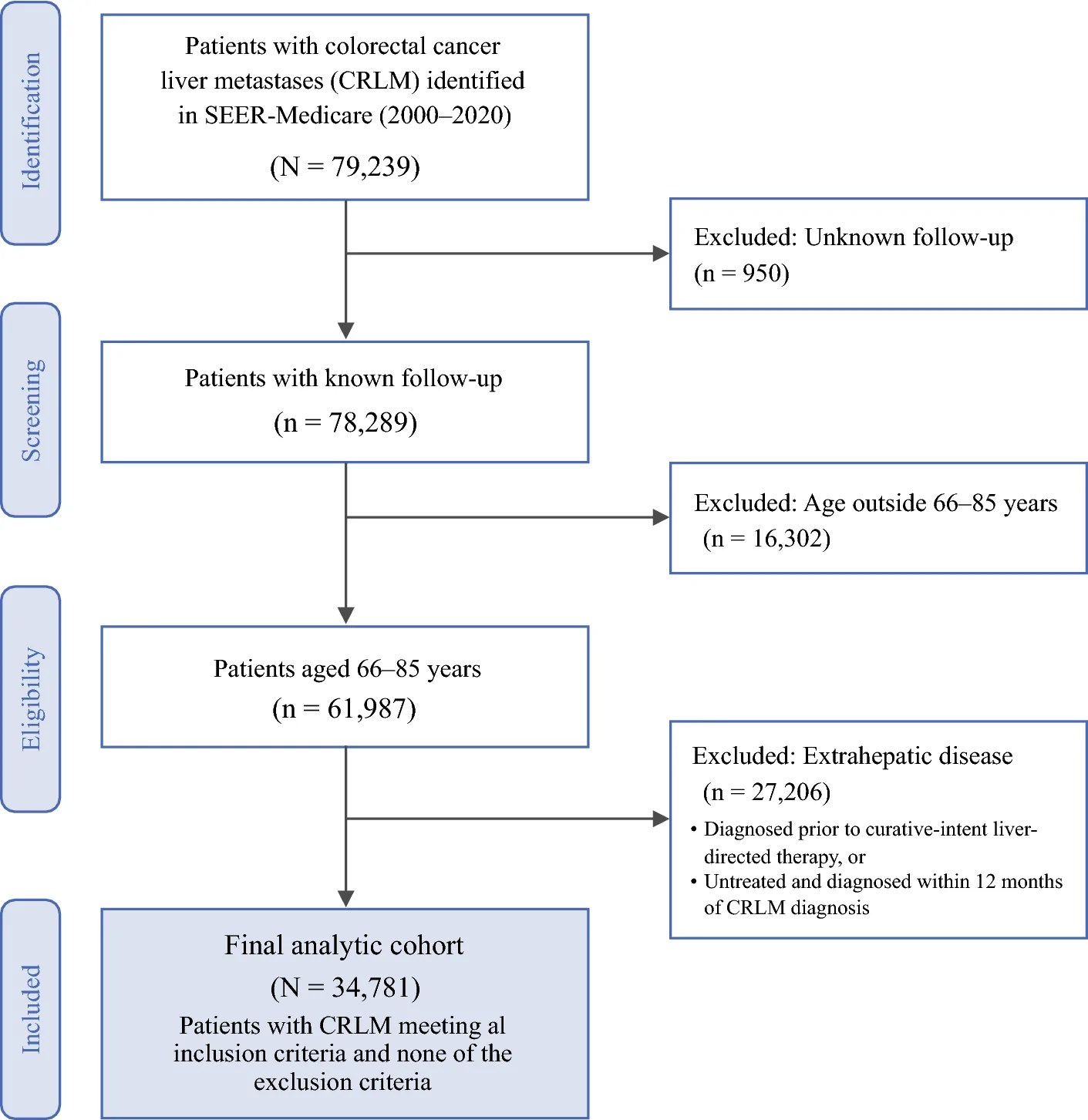
Consort diagram of cohort selection

A total of 79,239 patients with CRLM were initially identified. Patients with unknown follow-up (*n* = 950), those outside the age range of 66–85 years (*n* = 16,302), and those with extrahepatic disease (*n* = 27,206) were excluded, yielding a final analytic cohort of 34,781 patients (Fig. 1).

### Urban–Rural Classification

Residence was categorized using Rural–Urban Commuting Area (RUCA) codes. For the primary analysis, “urban” residence was retained as a distinct category, whereas “large rural city,” “small rural town,” and “isolated rural town” were combined into a single “rural” category to allow a binary comparison. In sensitivity analyses, the original RUCA classification was retained as a categorical variable to evaluate differences across rurality gradients.

### Covariates

Extracted demographic variables included age, gender, race/ethnicity, marital status, and socioeconomic status. Socioeconomic status was measured by using the Yost Index at the census-tract level and analyzed by quintiles, consistent with prior SEER-Medicare studies.^12–14^ Tumor-related variables included primary tumor site, histological differentiation, T-classification, N-classifications, and timing of metastatic disease presentation (synchronous vs metachronous). Treatment-related variables included primary tumor resection, systemic chemotherapy use, and receipt of curative-intent LDT (categorized as hepatectomy alone, ablation alone, hepatectomy with ablation, or liver transplantation). Time to liver-directed therapy was measured in months.

Vital status was obtained from the SEER database and supplemented with Medicare death dates for patients with missing or unknown SEER mortality information. Overall survival was defined from CRLM diagnosis to death from any cause, with censoring at last follow-up. Cancer-specific survival was not used given it is not available in the Medicare files.

### Statistical Analysis

Categorical variables were summarized by using frequency and percentages, and continuous variables by using means with standard deviations or medians with interquartile ranges. Univariable analyses used chi-squared test for categorical variables and the Student’s *t*-test for continuous variables. Multivariable logistic regression was used to identify demographic and tumor characteristics independently associated with urban/ rural residence. Distinct logistic regression models evaluated associations between rurality and receipt of primary tumor resection, systemic chemotherapy, and curative-intent LDT, adjusting for demographic and tumor characteristics.

Cox proportional hazards regression was used to evaluate the impact of rurality on overall survival. Socioeconomic status (Yost Index) was not included in the primary multivariable models because it is highly correlated with rural residence and may lie along the causal pathway between geographic residence and oncologic outcomes. Instead, socioeconomic status was evaluated in sensitivity analyses by using both categorical (quintiles) and collapsed binary (Q1-Q3 vs. Q4-Q5) specifications. A secondary Cox model adjusting for treatment variables (i.e., chemotherapy and LDT) was employed by using a 6-month landmark analysis to mitigate the immortal time bias. Patients who died or initiated treatment greater than 6 months from CRLM diagnosis were excluded from the landmark cohort. Additionally, an exploratory subgroup analysis was performed among patients receiving both curative-intent LDT and systemic chemotherapy. Overall survival was compared between rural and urban residents adjusting for the same covariates as the primary survival analysis. “Unknown” categories were retained for covariates with ≥ 30 observations to preserve sample size and reduce potential bias associated with listwise deletion of nonrandom missing data. All analyses were performed in Stata19 (Statacorp LLC; College Station, TX). Supplementary material 1 provides more detailed information on the statistical analysis.

### Ethical Aspects

This project was reviewed and approved by the Rutgers Institutional Review Board, which granted expedited review due to the use of de-identified registry data (Protocol #Pro2022001987).

### Role of the Funding Source

This work was supported by a generous grant from the Nussbaum Foundation (Cooperman Barnabas Medical Center, Livingston, NJ) and was used for biostatistical support. The funder did not play a role in the design of the study; the collection, analysis, and interpretation of the data; the writing of the manuscript; and the decision to submit the manuscript for publication. This study used the linked SEER-Medicaid database. The interpretation and reporting of these data are the sole responsibility of the authors. The collection of cancer incidence data used in this study was supported by the California Department of Public Health pursuant to California Health and Safety Code Section 103885; Centers for Disease Control and Prevention's (CDC) National Program of Cancer Registries, under cooperative agreement 1NU58DP007156; the National Cancer Institute's (NCI) SEER Program under contract HHSN261201800032I awarded to the University of California, San Francisco, contract HHSN261201800015I awarded to the University of Southern California, and contract HHSN261201800009I awarded to the Public Health Institute. The ideas and opinions expressed herein are those of the authors and do not necessarily reflect the opinions of the State of California, Department of Public Health, the NCI, and the CDC and Prevention or their Contractors and Subcontractors.

## Results

### Cohort Description

A total of 34,781 patients with isolated CRLM met the inclusion criteria (Table 1). Patient age was a mean 76 (SD 5.7) years, and the majority were male (54%), and non-Hispanic White (78.5%). Most patients lived in an urban geographic location (78.3%). Most patients had synchronous CRLM (65.8%).

**Table 1:** Patient clinicodemographics and their association with urban/rural status (*n* = 34,781)

	Residence		Univariable *P*-value	aOR	95% CI	Multivariable *P*-value
	Urban	Rural				
Age, years–mean (SD)	76.3 (5.0)	75.7 (5.0)	**< 0.001**	0.98	0.98, 0.99	**< 0.01**
*Gender*						
Male	14,629 (53.7)	2,973 (54.9)	**0.012**	**–**	**–**	**–**
Female	12,596 (46.3)	2,443 (45.1)	**-**	1.01	0.95, 1.07	0.74
*Race/ethnicity*						
White	20,846 (76.6)	4,783 (88.4)	**< 0.001**	**–**	**–**	**–**
Non-white	6,355 (23.3)	632 (11.7)	**–**	0.41	0.38, 0.45	**< 0.01**
Unknown	24 (0.1)	1 (0.0)	**–**	0.19	0.03, 1.42	0.11
*Marital status*						
Single	8,466 (31.1)	1,715 (31.7)	**< 0.001**	**–**	**–**	**–**
Partnered	9,880 (36.3)	2,393 (44.2)	**–**	0.93	0.87, 0.99	**0.02**
Unknown	8,879 (32.6)	1,308 (24.1)	**–**	7.37	6.54, 8.32	**< 0.01**
*Primary site*						
Right colon	9,999 (36.7)	1,984 (36.6)	**< 0.001**	**–**	**–**	**–**
Left colon	9,762 (35.9)	1,824 (33.7)	**–**	0.93	0.87, 1.00	0.06
Rectum	4,145 (15.2)	951 (17.6)	**–**	1.13	1.04, 1.24	**0.01**
Overlapping	3,319 (12.2)	657 (12.1)	**–**	1.03	0.94, 1.14	0.52
*Histologic differentiation*						
Well differentiated	1,435 (5.3)	310 (5.7)	0.153	**–**	**–**	**–**
Moderately differentiated	15,306 (56.2)	3,067 (56.6)	**–**	0.89	0.78, 1.01	0.07
Poorly differentiated	5,757 (21.1)	1,159 (21.4)	**–**	0.86	0.75, 0.99	**0.03**
Unknown	4,727 (17.4)	880 (16.2)	**–**	0.86	0.74, 0.99	**0.04**
*T-classification*						
T1-T2	8,768 (32.2)	2,013 (37.2)	**< 0.001**	**–**	**–**	**–**
T3-T4	3,055 (11.2)	681 (12.6)	**–**	0.98	0.89, 1.09	0.76
Unknown	15,402 (56.6)	2,722 (50.3)	**–**	0.88	0.81, 0.95	**< 0.01**
*N-classification*						
Node negative	7,312 (26.9)	1,609 (29.7)	**< 0.001**	**–**	**–**	**–**
Node positive	7,529 (27.7)	1,662 (30.7)	**–**	0.95	0.88, 1.04	0.26
Unknown	12,384 (45.5)	2,145 (39.6)	**–**	1.00	0.92, 1.10	0.93
*Metastatic disease*						
Metachronous	9,224 (33.9)	1,752 (32.4)	**0.029**	**–**	**–**	**–**
Synchronous	18,001 (66.1)	3,664 (67.6)	**–**	1.09	1.02, 1.17	**0.01**

For univariable analysis, categorical variables were analyzed with chi-squared tests and continuous variables with Student’s *t*-test. For multivariable analysis adjusted odds ratios (aOR) and 95% confidence intervals (CI) were estimated using logistic regression. Urban residence served as the reference category. All categorical variables were included with reference categories. *P*-values <0.05 are bolded. Percentages may not add up to 100% due to approximation

**Fig. 2:**
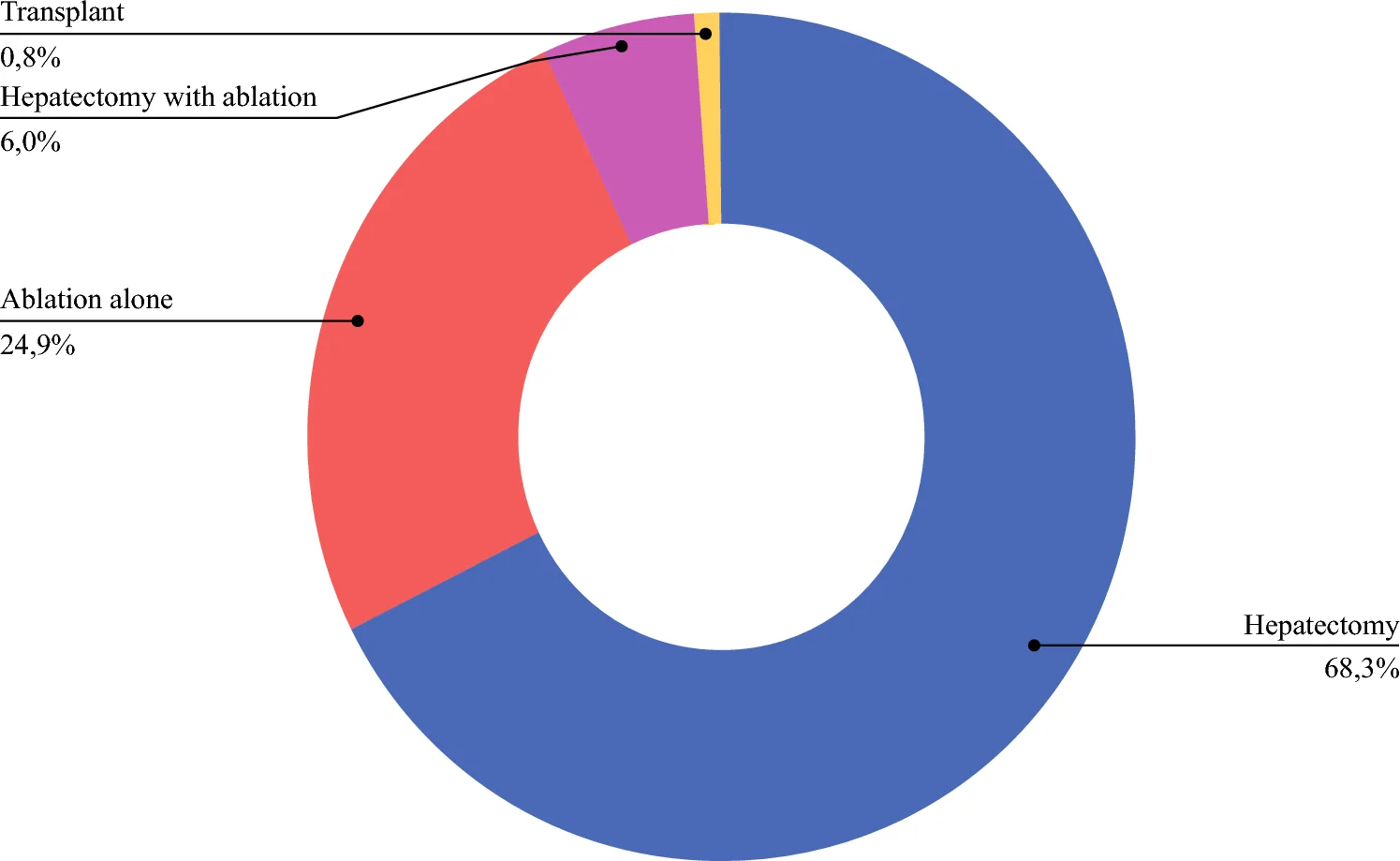
Type of curative-intent liver-directed therapy in patients with CRLM

A minority received curative-intent LDT (10.9%), most commonly hepatectomy without concurrent ablation (68.3%) (Fig. 2). The median (IQR) time from diagnosis to liver-directed therapy was 3 months (0–7 months). A majority of the patients in the cohort had a primary tumor resection (76.4%) and received systemic chemotherapy (63%).

### Association of Urban-Rural Status with Clinicodemographic and Treatment Variables

Several demographic and tumor differences between urban and rural patients were identified (Table 1). By multivariable logistic regression, rurality was independently associated with higher rates of primary tumor location in the rectum (adjusted odds ratio [aOR] [95% confidence interval (CI)] 1.13 [1.04–1.24], *p* = 0.01) and synchronous metastatic disease (aOR [95% CI] 1.09 [1.02–1.17], *p* = 0.01).

**Fig. 3:**
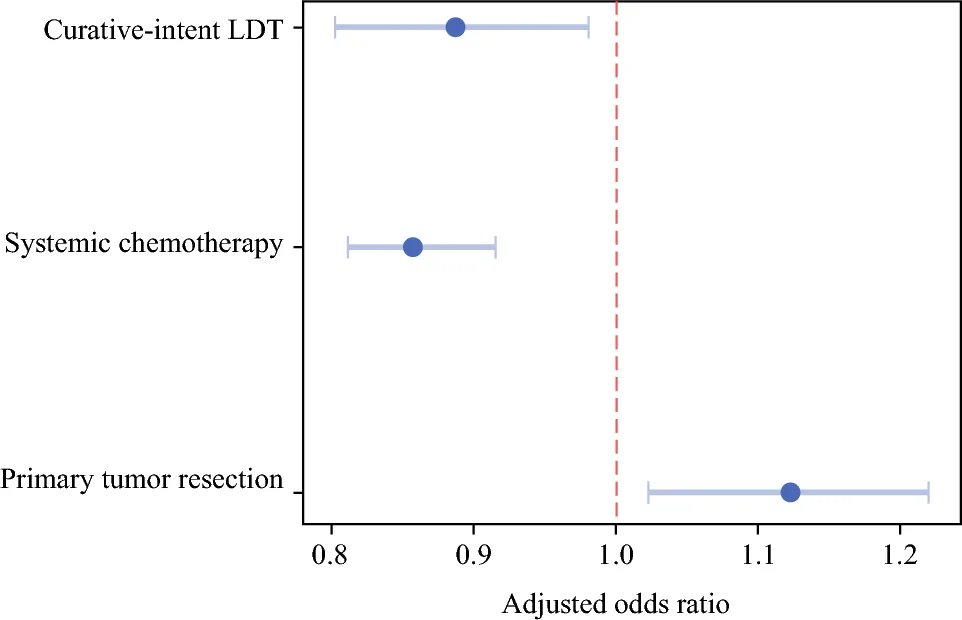
Forest plot of the association between rurality and treatment receipt in patients with CRLM. Points represent adjusted odds ratios (aOR) and error bars indicate 95% confidence intervals (CI). Vertical dashed line denotes the null value

Rural residence was associated with different treatment patterns (Fig. 3). Rurality was associated with lower rates of curative-intent LDT (aOR [95% CI] 0.89 [0.80–0.99], *p* = 0.03). Among patients who received LDT, the specific modalities did not differ by urban/rural status (Supplementary Material 2). Additionally, rural residence was associated with higher rates of primary tumor resection (aOR [95% CI] 1.12 [1.02–1.22], *p* = 0.01) yet lower rates of systemic chemotherapy (aOR [95% CI] 0.86 [0.81–0.92], *p* < 0.01). After adjustment, higher odds of primary tumor resection in rural patients were observed only in the synchronous cohort (Supplementary Material 3).

### Impact of Urban-Rural Status on Overall Survival

**Fig. 4:**
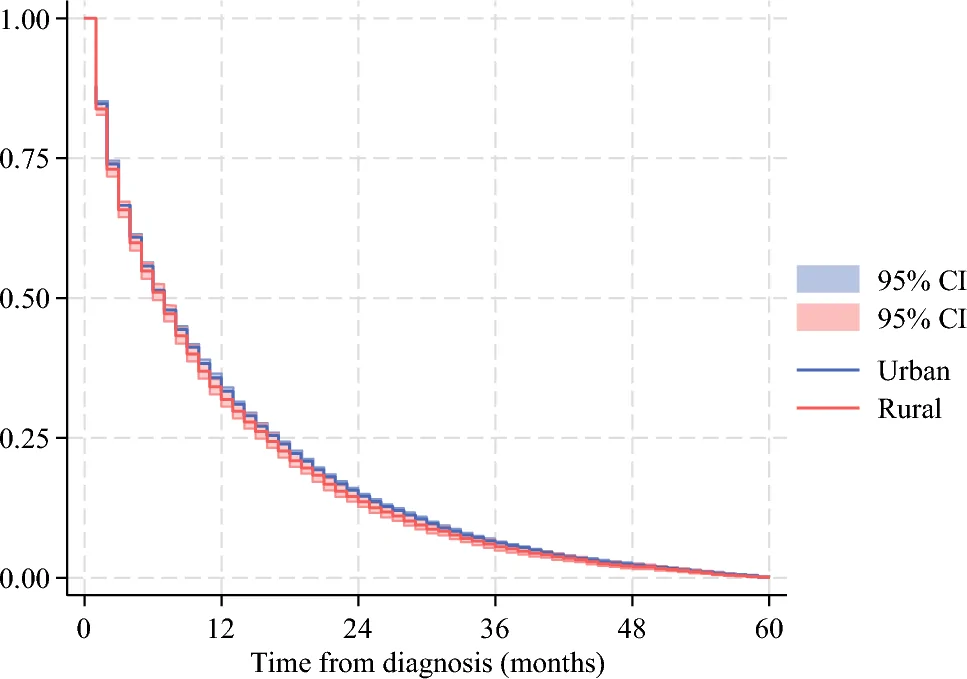
Kaplan–Meier estimates of overall survival with 95% confidence intervals in patients with CRLM, stratified by urban-rural residence. Curves are truncated at 60 months to enhance visualization of early differences

Patients were followed for a median 7 (IQR 2–20) months. The relatively short median follow-up reflects the advanced age and high early mortality in this cohort. Kaplan-Meier analysis with 95% CI demonstrated worse overall survival among rural patients compared with urban patients (Fig. 4). The survival impact of rurality was examined by using Cox proportional hazards modeling, adjusting for age, gender, race/ethnicity, marital status, primary tumor site, histologic grade, T classification, N classification, and type of liver metastasis. Rural residence was associated with a higher hazard of death (hazard ratio (HR) [95% CI] 1.08 [1.05–1.12], *p* < 0.01). Findings were consistent in sensitivity analyses adjusting for socioeconomic status by using the Yost Index (Supplementary Material 4). In a sensitivity analysis modelling RUCA as a categorical variable, findings were generally consistent across the rurality gradient (Supplementary Material 5). Although the magnitude and statistical significance of associations varied across individual rural categories, the overall direction of the treatment associations was consistent with the primary analysis. Compared with urban residence, all rural categories were independently associated with an increased risk of death.

Two subanalyses incorporating treatment strategy and using a 6-month landmark yielded consistent findings. First, adjusting for use of both chemotherapy and liver-directed therapy, rurality was associated with an increased hazard of death (HR [95% CI] 1.10 [1.05–1.14], *p* < 0.01). Second, adjusting for use of liver-directed therapy only, rurality remained associated with an increased risk of death (HR [95% CI] 1.10 [1.05–1.14], *p* < 0.01).

In an additional exploratory subgroup analysis restricted to patients who received both curative-intent LDT and systemic chemotherapy (*n* = 2,946), rural residence was not significantly associated with overall survival after adjustment (HR [95% CI] 1.13 [0.99–1.28], *p* = 0.07). However, the direction of association remained consistent with the primary analysis, with rural patients demonstrating a higher hazard of death compared with urban patients.

## Discussion

This study evaluated a national cohort of CRLM patients from the paired SEER-Medicare database to understand the independent impact of geographic rurality on disease presentation, treatment and survival for CRLM. Rural residence was associated with lower rates of systemic chemotherapy and curative intent LDT and inferior survival. These results highlight the geographic inequities in CRLM treatment and the need for focused strategies to improve access to complex oncologic CRLM care.

These findings reinforce the growing body of literature identifying area-level factors as a meaningful social determinant of access to curative-intent therapy for CRLM patients. Previous analysis of Medicare beneficiaries with isolated CRLM demonstrated only 8.5% receive LDT, with significantly lower odds for Black patients, and those from regions with higher social vulnerability and poverty.^7,15^ Each 10% increase in county poverty rate was independently associated with lower receipt of liver metastasectomy (odds ratio [OR] 0.82).^15^ Herein, we observed that rurality was associated with further disparities in the delivery of multidisciplinary cancer care. Even after controlling for patient and tumor related factors, rural residence was independently associated with lower odds of systemic chemotherapy and LDT use. These disparities likely reflect structural barriers inherent to rural healthcare systems, including limited availability of specialized cancer centers, hepatobiliary surgeons, multidisciplinary tumor boards, and coordinated referral pathways, as well as the travel burden associated with accessing tertiary care. In combination with the reduced availability of supportive oncologic services, these limitations create additional layers of difficulty for patients navigating complex treatment pathways. Patients in rural settings may face an increased burden of decision-making, often interpreting treatment options and coordinating care with limited specialist guidance, which can contribute to fragmented understanding of multimodality management and place disproportionate responsibility on patients in already resource-limited environments.^16^

The higher rate of primary tumor resection for rural patients in this study, despite the less frequent use of curative-intent liver-directed therapy, may reflect the fragmentation of oncologic care in rural regions. In particular, initial surgical management may occur locally, whereas referral for specialized LDT or multidisciplinary evaluation—typically located in more urban regions—remains inadequate. This disconnect between primary colon surgery and coordinated metastatic management has not been well characterized in prior CRLM-focused analyses. As such, our findings help address a knowledge gap identified in the literature regarding the mechanisms underlying rural-urban disparities in cancer outcomes.^11^

Third, rural residence was independently associated with a modest but significant decrease in overall survival among CRLM patients. Geographic disparities in cancer outcomes have been demonstrated across multiple settings, with rural populations experiencing lower all-cause and cancer-specific survival and progressively higher mortality with increasing remoteness.^17^ US-based analyses confirm similar trends in colorectal cancer, including reports of 11.8% higher mortality in rural counties compared with urban areas, a difference only partially mediated by socioeconomic conditions.^18,19^ In our analysis, adjustment for area-level socioeconomic status (modeled using Yost Index quintiles) modestly attenuated the association between rural residence and survival in sensitivity analyses, suggesting that area-level socioeconomic disadvantage contributes to, but does not fully explain, the observed survival gap. The persistence of the association after adjustment indicates that rural residence likely captures additional structural barriers to specialized cancer care. This observation aligns with findings by Molina et al., who demonstrated that county-level poverty was independently associated with lower receipt of liver metastasectomy among patients with CRLM, whereas no such association was observed for surgical management of stage I colorectal cancer.^15^ These findings emphasize that the combined influence of geography and socioeconomic context disproportionately affects access to complex cancer care. Supporting this structural explanation, Myers et al. reported that rural counties with higher proportions of residents lacking vehicles experienced particularly elevated mortality, highlighting how transportation barriers and geographic isolation can directly influence outcomes for patients requiring specialized treatment.^19^

Importantly, although adjustment of receipt of chemotherapy and LDT in the primary analysis did not fully eliminate the association between rural residence and overall survival, an exploratory analysis restricted to patients who received both LDT and systemic chemotherapy demonstrated an attenuated association that did not reach significance. These findings suggest that differences in access to and receipt of guideline-concordant multimodal therapy may contribute to the observed rural-urban disparity. However, the persistence of an elevated hazard of death after accounting for chemotherapy and LDT suggests that differences in treatment receipt alone do not fully explain the survival disparities observed. This may be explained by the small fraction of patients who received LDT and/or differences in tumor biology and metastatic burden. Rural residence was associated with a higher incidence of rectal primary tumors, which may be associated with higher rates of bilobar liver involvement and inferior outcomes following hepatectomy.^20,21^ These biological and anatomic differences, when combined with the structural barriers associated with rural healthcare delivery, may multifactorially contribute to the observed survival differences.

Several limitations warrant emphasis. Due to the retrospective nature of our work, our results measure associations between demographic factors and the treatments pursued; conclusions regarding the specific impact of these demographic and socioeconomic factors on treatment decisions should not be drawn. Additionally, our study population is limited to a subset of elderly US adults enrolled in Medicare and thus is not representative of the general US population.^22^ Third, although information regarding the patient’s disease status (i.e., tumor site, histological differentiation, staging, and timing of metastatic disease) was included in the adjusted analyses, several additional data were not available in SEER, including both clinical factors (tumor burden and distribution of CRLM, KRAS mutation status) and treatment factors (treatment-related toxicities, surgical margin).^23,24^ Therefore, we cannot exclude the possibility that differences in metastatic burden or resectability contributed to observed rural-urban differences in treatment patterns and outcomes. Cancer-specific survival could not be assessed in this dataset because it was not available in Medicare files; therefore, OS was used as the primary endpoint. Additionally, extrahepatic metastatic disease was identified using Medicare claims because this information is not directly captured in SEER. Although exclusion of patients with extrahepatic disease prior to curative-intent LDT or within 12 months of CRLM diagnosis was intended to reduce misclassification of occult extrahepatic metastatic disease and was consistent with prior SEER-Medicare studies of CRLM, claims-based identification may introduce residual misclassification and selection bias.^12,13^ Fourth, the lack of travel distance precluded analysis to determine the relative contribution of rurality on increasing travel distance to specialized centers. Although socioeconomic status was measured via the Yost Index and RUCA codes were used to define rurality, residual geographic heterogeneity may persist across states, and we did not include state-level fixed effects or multilevel modeling. Travel distance is associated with lower odds of LDT^7^ and more than 40% of Medicare patients reside in health service areas where no liver surgery is performed,^13^ highlighting the need for significant travel for most CRLM patients. Relatedly, the “rural” cohort included individuals from larger rural cities, small rural towns, and isolated rural towns, which may have masked heterogeneity in treatment decisions across rural subgroups. Patients living in more remote areas may face even greater transportation and geographic barriers to specialized healthcare.^25^ Lastly, care that was paid for via entities outside of Medicare are not included in the SEER database.^26^ Given that rural status may be associated with higher rates of self-pay, patients’ full treatment plan may not be represented in our analysis.

## Conclusions

Despite similar tumor characteristics and higher likelihood of primary tumor resection, rural patients with CRLM were less likely to receive LDT and experienced worse survival. These findings highlight persistent geographic disparities in cancer care delivery and outcomes, underscoring the need for targeted system-level interventions specific to rural populations. Previous efforts have demonstrated the success of virtual multidisciplinary tumor boards, telemedicine-based care models, regional referral networks through hub-and-spoke models, and community outreach and education as available tools to address this critical unmet need to improve LDT utilization and survival for rural populations.^27–30^

## Supplementary Information

Below is the link to the electronic supplementary material.

Supplementary file1 (DOCX 10 KB)

Supplementary file2 (DOCX 8 KB)

Supplementary file3 (DOCX 8 KB)

Supplementary file4 (DOCX 8 KB)
