# Supplementary material for: Urban-Rural Disparities in Treatment and Outcomes for Colorectal Liver Metastases in the United States: A SEER-Medicare Analysis
Source: Ann Surg Oncol. 2026 Aug 28;33(12):10029–37. doi: 10.1245/s10434-026-20434-5 (PMC13675326; doi:10.1245/s10434-026-20434-5)
Supplement: Supplementary file 1 [file 10434_2026_20434_MOESM1_ESM.docx]

**Supplementary material 1.** Supplementary statistical methods.

Variables available in the queried database were purposefully selected for inclusion in the univariable and multivariable analyses. Independent demographic variables included patient age, gender (male or female), race/ ethnicity (non-Hispanic White, non-Hispanic Black, Hispanic, Asian/ Pacific Islander, American Indian, or unknown), and marital status (single, partnered, or unknown). Independent tumor characteristics included primary tumor site (right colon, left colon, rectum, or overlapping), histologic grade (well, moderately, or poorly differentiated, or unknown), T classification (T1, T2, T3, T4, or unknown), N classification (node negative, node positive, or unknown), and type of liver metastasis (synchronous or metachronous).

For analytic purposes, race/ ethnicity was re-coded into three categories: “White”, “non-White” and “unknown”. The “non-White” category encompassed non-Hispanic Black, Hispanic, Asian/ Pacific Islander, and American Indian patients. Similarly, T classification was grouped into three categories: “T1-T2”, “T3-T4” and “unknown”To construct the urban-rural residence variable, the categories “large rural city”, “small rural town”, and “isolated small rural town” were combined into a single “rural” category. Observations labeled as “unknown” for residence were excluded to yield a dichotomous “urban” versus “rural” variable. Urban residence was used as the reference category in all regression analyses as it reflected the largest and most well-characterized group in the dataset.

Socioeconomic status was measured using the Yost Index and included as a contextual socioeconomic variable. Given the conceptual overlap between area-level socioeconomic status and geographic residence, and the potential for socioeconomic factors to lie on the causal pathway between rurality and treatment access and outcomes, the Yost Index was evaluated in sensitivity analyses rather than included in the primary multivariable models. This approach allowed assessment of the robustness of the rurality outcome association across alternative adjustment sets. The Yost Index was analyzed in descriptive analyses to evaluate the distribution across urban and rural residence. In sensitivity analyses, the Yost Index was modeled in two forms: as a categorical variable using quintiles, and as a collapsed binary variable (Q1-Q3 vs Q4-Q5).

Dependent treatment variables included primary tumor resection, systemic chemotherapy, and curative-intent liver therapy. Each of these was treated as dichotomous. A categorical variable describing the type of curative-intent LDT (hepatectomy, ablation alone, hepatectomy with ablation, or transplant) was also analyzed. Overall vital status (alive or deceased) was used as the dependent variable in survival analyses.

Reference categories for the remaining categorical variables were selected to facilitate clinical interpretability and provide a meaningful baseline. “Male” and “White” were chosen as the reference for gender and race/ ethnicity, respectively, reflecting the majority groups in the cohort. “Single” was used as the reference for marital status to compare partnered patients. For tumor characteristics, “right colon”, “well-differentiated”, “T1”, “node-negative”, and “metachronous” were used as references, representing the most common (right colon) or least aggressive categories (all others). For treatments, “no” was used as the reference for dichotomous variables, and “hepatectomy” was used as the reference for type of curative-intent liver therapy, as it represents the reference standard due to its established superiority in overall survival and disease-free survival, especially for technically resectable disease. [1, 2]

Univariable analyses were performed to examine associations of urban-rural residence with demographic and tumor characteristics, treatments, and outcomes. Chi-squared test for categorical variables and the Student’s *t*-test for the only parametric continuous variable (age).

Multivariable analyses were performed in two domains. First, logistic regression was used to assess demographic and tumor characteristics independently associated with urban-rural residence. Second, the impact of urban-rural residence on treatment receipt was assessed. Logistic regression was used to evaluate dichotomous treatment outcomes (primary tumor resection, systemic chemotherapy, and curative-intent liver therapy), adjusting for demographic and tumor characteristics.

A subanalysis was performed to evaluate primary tumor resection specifically within the metachronous and synchronous metastases subgroups. This involved repeating the logistic regression models for primary tumor resection separately in each subgroup, adjusting for the same demographic and tumor characteristics used in the overall multivariable analyses.

Cox proportional hazards regression was used to evaluate overall survival, adjusting for the same covariates. A subanalysis was performed to evaluate survival after additional adjustment for chemotherapy and liver-directed therapy. For this subanalysis, a landmark analysis was performed, excluding patients who did not survive or did not receive treatment within the first six months after CRLM diagnosis. This approach mitigated immortal time bias. The landmark analysis was considered a secondary analysis rather than part of the primary survival models because it conditions on survival beyond the six-month landmark, thereby addressing a distinct research question and estimating conditional, rather than overall survival from diagnosis. Primary tumor resection was not adjusted for, as it has not been shown to influence survival in this patient population. [3]

**References**

1. van der Lei S, Puijk RS, Dijkstra M, et al. Thermal ablation versus surgical resection of small-size colorectal liver metastases (COLLISION): an international, randomised, controlled, phase 3 non-inferiority trial. Lancet Oncol. 2025 Feb;26(2):187-199. doi: 10.1016/S1470-2045(24)00660-0.
2. Kron P, Linecker M, Jones RP, Toogood GJ, Clavien PA, Lodge JPA. Ablation or Resection for Colorectal Liver Metastases? A Systematic Review of the Literature. Front Oncol. 2019 Oct 16;9:1052. doi: 10.3389/fonc.2019.01052
3. Rahbari NN, Biondo S, Frago R, et al. SYNCHRONOUS and CCRe-IV Trial Groups. Primary Tumor Resection Before Systemic Therapy in Patients With Colon Cancer and Unresectable Metastases: Combined Results of the SYNCHRONOUS and CCRe-IV Trials. J Clin Oncol. 2024 May 1;42(13):1531-1541. doi: 10.1200/JCO.23.01540.
