# Supplementary material for: Urban-Rural Disparities in Treatment and Outcomes for Colorectal Liver Metastases in the United States: A SEER-Medicare Analysis
Source: Ann Surg Oncol. 2026 Aug 28;33(12):10029–37. doi: 10.1245/s10434-026-20434-5 (PMC13675326; doi:10.1245/s10434-026-20434-5)
Supplement: Supplementary file 2 [file 10434_2026_20434_MOESM2_ESM.docx]

**Supplementary material 2.** Patient curative-intent LDT modalities by urban/rural status (n=3,040).

|  | **Residence** | | **Univariable P-value** |
| --- | --- | --- | --- |
| **Curative intent liver therapy** | Urban | Rural | 0.141 |
| Hepatectomy | 1,698 (66.5) | 326 (67.1) |  |
| Ablation alone | 694 (27.2) | 119 (24.5) |  |
| Hepatectomy with ablation | 139 (5.4) | 38 (7.8) |  |
| Transplant | 23 (0.9) | 3 (0.6) |  |

**Caption:** Categorical variables were analyzed with chi-squared tests. Percentages may not add up to 100% due to approximation.
