# Supplementary material for: Urban-Rural Disparities in Treatment and Outcomes for Colorectal Liver Metastases in the United States: A SEER-Medicare Analysis
Source: Ann Surg Oncol. 2026 Aug 28;33(12):10029–37. doi: 10.1245/s10434-026-20434-5 (PMC13675326; doi:10.1245/s10434-026-20434-5)
Supplement: Supplementary file 3 [file 10434_2026_20434_MOESM3_ESM.docx]

**Supplementary material 3.** Primary tumor resection by metastases type and urban/ rural status

| **Metastatic disease** | **Urban** | **Rural** | **P-value** | **aOR** | **95% CI** | **P-value** |
| --- | --- | --- | --- | --- | --- | --- |
| Metachronous | 8,345 (83.7) | 1,626 (16.3) | **0.002** | 1.17 | 0.94-1.45 | 0.15 |
| Synchronous | 12,118 (82.5) | 2,572 (17.5) | **0.001** | 1.12 | 1.02-1.24 | **0.02** |

**Caption:** Percentages represent the proportion of urban/ rural patients within each metastasis subtype among those who underwent primary tumor resection. Adjusted odds ratios (aOR) and 95% confidence intervals (CI) were estimated using logistic regression. Urban residence served as the reference category. P-values <0.05 are bolded.

In the overall cohort, rural patients had slightly higher odds of undergoing primary tumor resection compared with urban patients (aOR [95%CI]: 1.12 [1.02-1.22], p=0.01). To further explore this finding, we evaluated resection by metastases type. The primary tumor resection rate in patients with metachronous and synchronous metastases was 91.0% and 68.8%, respectively. In univariable analysis among patients in the metachronous and synchronous groups, rural patients were less likely to undergo primary resection (83.7% urban and 16.3% rural, p=0.001 and 82.5% urban and 17.5% rural, respectively). However, after adjustment, only patients with synchronous metastases residing in rural settings were significantly more likely to undergo resection (aOR [95%CI]: 1.12 [1.02-1.24], p=0.02). Overall, these findings suggest that the higher primary resection rates observed in rural patients in the full cohort are not uniform across metastases type, and that in synchronous disease, rurality itself is independently associated with higher resection after accounting for patient and tumor characteristics. The overall rural effect is driven by synchronous disease.
