# Supplementary material for: Urban-Rural Disparities in Treatment and Outcomes for Colorectal Liver Metastases in the United States: A SEER-Medicare Analysis
Source: Ann Surg Oncol. 2026 Aug 28;33(12):10029–37. doi: 10.1245/s10434-026-20434-5 (PMC13675326; doi:10.1245/s10434-026-20434-5)
Supplement: Supplementary file 4 [file 10434_2026_20434_MOESM4_ESM.docx]

**Supplementary material 4.** Multivariable analysis of impact of rural residence on treatment receipt and overall survival (urban reference) with additional adjusting for socioeconomic status measured by the Yost Index in categorical (Q1-5) and collapsed (Q1-3 versus Q4-5) versions.

|  | **Yost Index categorical** | | | **Yost Index collapsed** | | |
| --- | --- | --- | --- | --- | --- | --- |
|  | OR/aHR | 95% CI | P-value | aOR/aHR | 95% CI | P-value |
| **Primary tumor resection** |  |  |  |  |  |  |
| No | - | - | - | - | - | - |
| Yes | 1.20 | 1.10, 1.32 | **<0.01** | 1.20 | 1.09, 1.31 | **<0.01** |
| **Systemic chemotherapy** |  |  |  |  |  |  |
| No | - | - | - | - | - | - |
| Yes | 0.94 | 0.88, 1.01 | 0.09 | 0.93 | 0.87, 0.99 | **0.03** |
| **Curative intent liver therapy** |  |  |  |  |  |  |
| No | - | - | - | - | - | - |
| Yes | 0.95 | 0.85, 1.06 | 0.36 | 0.94 | 0.85, 1.05 | 0.28 |
| **Overall survival** | 1.04 | 1.01, 1.08 | **0.02** | 1.05 | 1.01, 1.08 | **0.01** |

**Caption:** Adjusted odds ratios (aOR) and 95% confidence intervals (CI) were estimated using logistic regression for categorical variables. Categorical variables were presented with reference categories. Adjusted hazards ratio (aHR) and CI were estimated using Cox proportional hazards regression models. Urban residence served as the reference category. P-values <0.05 are bolded
